# Influence of ventilation use and occupant behaviour on surface microorganisms in contemporary social housing

**DOI:** 10.1038/s41598-020-68809-2

**Published:** 2020-07-16

**Authors:** T. Sharpe, G. McGill, S. J. Dancer, M.-F. King, L. Fletcher, C. J. Noakes

**Affiliations:** 1 Department of Architecture, University of Strathclyde, 75 Montrose Street, Glasgow, G1 1XJ Scotland, UK; 2 Dept. of Microbiology, Hairmyres Hospital, NHS Lanarkshire, Glasgow, Scotland, UK; 3 School of Applied Sciences, Edinburgh Napier University, Edinburgh, Scotland, UK; 4 Water, Public Health and Environmental Engineering Group, School of Civil Engineering, University of Leeds, Leeds, UK

**Keywords:** Public health, Bacteria, Microbial communities, Engineering

## Abstract

In the context of increasingly airtight homes, there is currently little known about the type and diversity of microorganisms in the home, or factors that could affect their abundance, diversity and nature. In this study, we examined the type and prevalence of cultivable microorganisms at eight different sites in 100 homes of older adults located in Glasgow, Scotland. The microbiological sampling was undertaken alongside a household survey that collated information on household demographics, occupant behaviour, building characteristics, antibiotic use and general health information. Each of the sampled sites revealed its own distinct microbiological character, in both species and number of cultivable microbes. While some potential human pathogens were identified, none were found to be multidrug resistant. We examined whether the variability in bacterial communities could be attributed to differences in building characteristics, occupant behaviour or household factors. Sampled sites furnished specific microbiological characteristics which reflected room function and touch frequency. We found that homes that reported opening windows more often were strongly associated with lower numbers of Gram-negative organisms at indoor sites (p < 0.0001). This work offers one of the first detailed analysis of cultivable microbes in homes of older adults and their relationship with building and occupancy related factors, in a UK context.

## Introduction

Homes satisfy our most basic needs for shelter and should be designed to provide a comfortable and safe environment. However, provision of housing is constrained through cost and legislation encompassing appearance, structure, materials, provision of services and energy performance. Most of the time these align to human needs, but there are conflicts, particularly with respect to energy and ventilation and their influence on health. In a bid to reduce energy and carbon emissions, the building sector is delivering increasingly airtight homes that aim to reduce uncontrolled ventilation losses^1^. There are concerns that without improved designed ventilation provision this strategy may lead to a range of unintended consequences including impacts on occupant health^2^. Ventilation affects exposure to a number of elements that are known to influence health, including chemicals, moisture, temperature and microorganisms. There is evidence that poor ventilation may be linked with poor physical and mental health in a number of non-domestic building types^3^, but whilst the literature points to detrimental effects in housing^2,4^, this remains seriously under investigated. In particular, there are currently gaps in knowledge about the range and diversity of microorganisms in the domestic environment, particularly in the context of modern airtight homes^5^. People spend a great deal of time in their homes, especially those at the extremes of age, and therefore the indoor microbiome could impact upon human health in ways not yet understood^6^.

Recent research into the real world performance of buildings has begun to reveal significant performance gaps in environmental conditions, especially poor rates of ventilation, particularly in bedrooms^7^. This has led to studies that have examined the consequences of increasing airtightness of modern construction, lack of ventilation, occupant interaction with ventilation, and increasing use of mechanical ventilation^8,9^. Recent reviews in the UK by the Royal College of Paediatrics and Child Heath^10^ and the National Institute for Health and Care Excellence^11^ have undertaken systematic reviews of the literature and recognised the importance of indoor air quality on health and the need to address challenges including with building design. Making buildings resistant to heat loss and draughts has important benefits; higher levels of insulation and airtightness can improve health, for example, through a reduction in cold-related deaths, condensation and mould indoors and reduced fuel poverty^12,13^. However, one of the consequences is a separation from the outdoor environment and lower ventilation rates. Theoretical analysis of housing types indicates that potential health consequences of reduced ventilation may include transmission of infectious diseases^14^, and there is emerging evidence that building design affects indoor microflora, with artificial environments created by mechanical ventilation having less diverse microbial communities with a higher presence of pathogens^15,16^. Whilst a small number of US studies have demonstrated that architectural design features (such as spatial arrangement or room type) can have an impact on the microbial biogeography of buildings, it remains unclear whether generalizable patterns exist that can be used to inform practice (e.g. through ‘bio-informed’ design)^17^. Moreover, the impact of improved thermal performance (and comfort) standards, energy conservation measures^18^ and the creation of hygrothermally stable indoor environments in contemporary housing^8^ on indoor microbiology have yet to be fully understood.

There is growing evidence that both building design and human behaviour determine the microbial species present in homes. Care homes have been shown to be a reservoir for antibiotic resistant bacteria including *Klebsiella* spp. and *E. coli*^19^, and environmental sampling has shown that high-touch surfaces in home environments may harbour methicillin-resistant *Staphylococcus aureus* (MRSA)^20^. Multi-drug resistance is having a huge impact in hospitals worldwide but is not commonly investigated in people’s homes; exceptions are the studies by Lax et al., who investigated antimicrobial resistance (AMR) in a hospital setting as well as private homes^21,22^. Use of antimicrobial cleaning products have also been highlighted as an emerging AMR risk factor in community settings^23^ including use of microbiocidal products used for routine cleaning following confirmed links between disinfectants and resistance^24^. There has even been a call for regulatory use of these products in hospitals^25^; powerful disinfectants harm the surface ecology, much like antibiotics harm healthy gut flora, permitting naturally tolerant or resistant microflora to survive and create reservoirs of increasingly resistant microbes^26,27^.

A number of previous studies have applied DNA sequencing methods to demonstrate the huge diversity in microorganisms present in the built environment^28^. Microbial control and building confinement are known to affect the composition and functional capabilities of the residing microbiome^26^. For example, fungi isolated from home surfaces tend to reflect species found in outside air (where there is no significant moisture damage in the building envelope), while bacterial contamination is more likely to derive from colonised inhabitants and/or their practices^28^; this suggests that ventilation provision and use may influence the fungal content of the home microbiome. Studies show that the microbial community in a building is influenced by several factors including location and climate^29^, occupant presence and behaviour (including antibiotic use)^30^, presence of pets^28^ and ventilation approach^31^. They have also shown that mechanically ventilated buildings have a lower microbial diversity than those that are naturally ventilated, and that the microorganisms present in mechanically ventilated buildings are dominated by human related species, with a much lower presence of environmental species^16^.

Exposure to a wide diversity of microbes has been found to confer protection against certain diseases which has led researchers to suggest that mechanical ventilation may be altering the microbial balance in a building, and could lead to “selection” of microorganisms that are more likely to cause disease or allergies^15^. However, the majority of these studies are conducted outside the UK, with a large proportion in US homes which have different construction, ventilation and climatic conditions. Few studies have performed systematic screening of sites in social housing in order to provide cultivable microorganisms, with a view to establishing the presence of human pathogens and AMR. As drivers for energy reduction have led to buildings becoming more airtight, with reduced ventilation rates and increasing use of mechanical ventilation, insight into the potential consequences of these measures is needed. Given the changes in housing design and construction we need to understand whether our approaches might encourage environmental persistence of a range of pathogens, and evidence from a UK context to support guidance and practice is required.

This study examined methodologies for assessing surface microorganisms in homes and investigated relationships between microorganisms and building characteristics to better understand how they may be affected by the design and use of buildings. The hypothesis is that with reduced ventilation and interaction with the external environment, there will be less diversity in the indoor microbiome and certain organisms may predominate. Whilst ventilation has been identified as a primary driver in the structure of the microbial community in buildings^16,31,32^, the impact of ventilation type, effectiveness and operation warrants further investigation^33^. In particular, the aim is to examine whether ventilation use leads to a change in the persistence of microorganisms, and to explore both design and lifestyle to assess potential reasons for this.

The study was conducted in two phases, the first of which conducted a household survey and microbial sampling of 100 homes and is reported here. The second undertook more detailed monitoring and analysis of 21 selected homes. We systematically screened specific sites in suburban social housing in order to determine the amount and type of cultivable aerobic bacteria and fungi at key sites in the home. Microbiological data is compared against responses to an extensive household questionnaire containing occupant and housing information. Through this we explore a range of different ventilation and occupancy factors that influence the indoor microbiology of homes and may potentially have an impact on occupant health. We also discuss research methods and protocols that could be applied to larger studies.

## Results

### Building and occupancy characteristics

The age of the homes ranged from 1995 to 2017, with 34% constructed pre-2010, when building regulations were revised to require airtightness reporting (see Table 1). The majority had either one or two bedrooms (94%), with 5% reporting three bedrooms and 1% reporting four or more. Where data was available, air tightness levels ranged from 2.96 to 7.3 m^3^/h/m^2^. Airtightness is important in terms of the context for this study as airtight dwellings are entirely reliant on the ventilation provision, and its use by occupants.

**Table 1:** Building and ventilation characteristics of sampled homes in and around Glasgow.

Development code	Ventilation type	Build year	Typology	No. beds	Airtightness (m^3^/h/m^2^)	No. homes surveyed
BS	Intermittent	1998	Flats	1 bed	n/a	6
CC	Intermittent	2000	Flats	1 bed	n/a	5
CG	Intermittent	2013	Cottages	2 bed	7.3	5
DR	Intermittent	2016	Flats/terraced	1–3 bed	4.72	13
FR	Intermittent	2013	Flats	2 bed	5.39	6
HC	Intermittent	2010	Cottages	1 bed	4.15	1
KP	Intermittent	1995	Flats	1–3 bed	n/a	5
KC	Intermittent	2009	Flats	2 bed	n/a	10
LA	dMEV	2017	Flats	2 bed	4.65	17
LR	Intermittent	2009	Flats	2 bed	n/a	1
LC	MVHR	2017	Flats/terraced	1–3 bed	2.96	11
MB	dMEV	2016	Flats	1–2 bed	4.68	5
MN	Intermittent	2003	Flats/cottages	1–2 bed	n/a	5
MP	Intermittent	2011	Flats	2 bed	5.53	6
NR	Intermittent	2010	Terraced	2 bed	n/a	2
MS	EAHP	2010	Flats/terraced	2–3 bed	n/a	6
WC	Intermittent	2016	Flats	2 bed	4.68	5
						109

*n/a* not available.

### Occupancy

The majority of homes were occupied by one or two persons (91%), with a small number of three (2%), four (6%) and five (1%) person households. The reported prevalence of smoking among surveyed households (29.4%) was slightly higher than the Scottish average of 21%^34^. 94% of households had at least one person over the age of 50.

Occupancy levels did not vary much between times of the day or days of the week and show consistent occupancy, suggesting relatively stable indoor conditions. The majority of households were typically occupied during the weekday by one (65%), two (22%) or three (2%) persons. Higher occupancy levels of four (6%) or five persons (1%) were reported in a small number of homes during the evening and at night. Of the 109 homes who completed the household survey, 25% of households reported the presence of pets, including dog(s) (18%) and cat(s) (5%). In 3% of households, pets (all dogs) had been prescribed antibiotics in the last six months.

Respondents were asked about recent antibiotic use, general health and recent hospital exposure. 60% of households reported visiting a hospital, doctor’s surgery or clinical environment in the month prior to the survey, 17% in the previous week. 38% of households reported taking antibiotics in the last year mostly to treat chest infections (19%), with a small number of households (5%) reporting antibiotic use in the last month. Of those who reported taking antibiotics, 98% stated that they completed the full course. A high percentage of respondents reported health conditions including arthritis (41%), respiratory disease (28%), diabetes (14%), and heart disease (17%).

The majority of households reported brushing floors (73%), dusting (75%) and vacuuming (74%) on a weekly basis. Most respondents cleaned the homes themselves (82%), although 8% of homes used a cleaning service/cleaner. 97% of households reported using antibacterial cleaning agents including disinfectants, the most common being anti-bacterial surface sprays, washing-up liquid and wipes. Over half of homes (51%) reported using bleach to clean their home. The majority of homes (96%) reported using an antibacterial cleaning product in the home in the week prior to sampling.

### Ventilation

Of the surveyed homes, the majority (64%) used natural ventilation (windows and trickle vents) and intermittent (controlled by manual use, humidistat control or lighting) mechanical extract fans located in kitchens and bathrooms. Whole house mechanical ventilation with heat recovery (MVHR) systems was installed in 10% of homes. A small number of homes (6%) utilised an exhaust air heat pump system (EAHP), which extracts air from rooms via ventilation ducts, with background ventilation provided by wall mounted vents. 20% of homes were ventilated with decentralised mechanical ventilation (dMEV) which provides low level continuous extract from kitchens and bathrooms with make-up air provided by trickle vents.

Window opening frequency can influence the prevalence of human and outdoor-associated microorganisms present in the indoor environment^32^. The results from the household survey indicate that almost half of households open windows on a daily basis during winter. Daytime window opening was much more prevalent than night-time. Window opening was found to be most prevalent in bedrooms, followed by living rooms and kitchens. The most predominant barrier to window opening was weather (73%), followed by heat loss (42%) and cold draughts (40%), suggesting window opening behaviour was dominated by thermal comfort as opposed to air quality considerations. The duration of window opening is not known. However, is it more likely that window opening for thermal comfort would be time limited, being driven by adaptive comfort, whereas window opening in bedrooms at night would be continuous overnight, albeit as a smaller aperture.

### Bedroom conditions

The bedroom environment is of considerable interest as people spend one-third of their lifetime in their bedroom, with time-use studies suggesting this may be higher for older adults^35,36^. It is estimated that exposure to indoor air pollution may be up to 16 times higher in the bedroom compared to the rest of the home^36^. Bedrooms with doors closed for privacy and windows closed for energy conservation are often poorly ventilated^37^, with studies highlighting poor bedroom ventilation in modern Scottish homes^38,39^. In addition, bedrooms overnight typically present steady-state conditions with limited adaptive behaviour, which can be useful when examining the effects of ventilation where confounding factors are minimised.

Reported occupancy in the main bedroom varied from one (70%) to two adults (30%). The majority of second bedrooms (where present), were occupied by a single adult, however 5% of homes reported children present. Overall, 19% of households stated that they normally open their bedroom window(s) at night during the winter. A further 4% of households reported opening their bedroom window on a weekly basis at night. All households reported closing curtains/blinds at night with 46% also reporting closing the bedroom door. This could have implications on the effectiveness of ventilation strategies due to the occlusion of trickle vents by curtains or blinds, or the obstruction of internal ventilation pathways by the closing internal doors^39^.

### Microbial results

**Figure 1:**
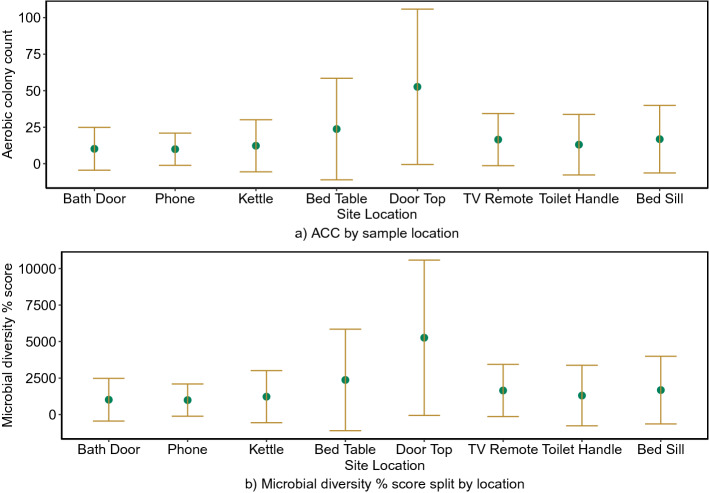
Mean and standard deviation of microorganisms across the eight sample locations over all homes.

Sampling sites covered a range of locations that were considered to be high touch (bathroom door handle, kettle handle, phone, toilet flush handle, TV remote) and lower touch where environmental contamination may be more important (bedside table, windowsill, door top). Each site presented specific microbiological characteristics which reflected the room function and touch frequency. Most sites yielded a mixture of coagulase-negative staphylococci, *Bacillus* spp., and micrococci, with occasional filamentous fungi and yeasts. Summary statistics of Aerobic Colony Count (ACC) on nutrient agar across all 100 homes are given in Table 2, and Fig. 1 shows the distribution of ACC and microbial diversity for the different sites.

**Table 2:** Summary statistics of log_10_ ACC on nutrient agar categorised by sample location.

Site	Mean (ACC) (n = 100)	Standard deviation (ACC) (n = 100)
Bathroom door handle (bathroom)	9.5	14.3
Phone^a^	9.6	11.0
Kettle (kitchen)	11.7	17.6
Bedside table (bedroom 1)	23.4	34.6
Door top (bedroom 1)	52.6	53.2
TV remote^a^	16.3	17.9
Toilet flush handle (bathroom)	12.0	20.2
Window sill (bedroom 1)	16.6	23.1

^a^Phone and TV remote were located at various sites in the home.

**Figure 2:**
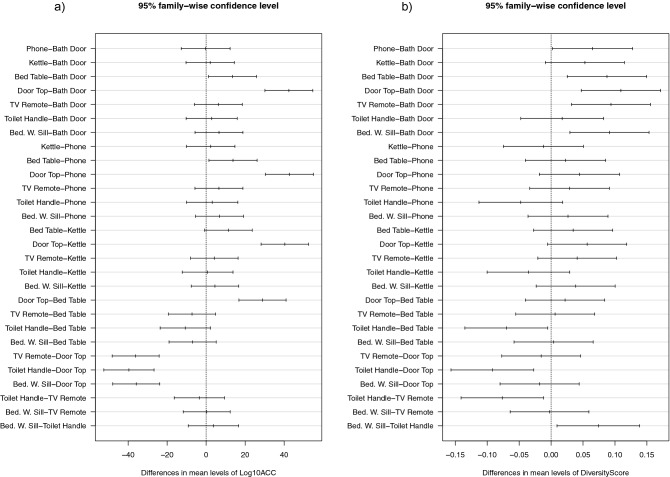
Mean difference plots representing Tukey HSD post-hoc comparisons between sites. Comparisons that do not pass through 0 are significantly different.

Post-hoc analysis shows significant differences of ACC between certain pairs of sample sites and not others (see Fig. 2). Most notably the ACC counts on the door top are higher and significantly different to all other surfaces, whereas ACC from frequently touched surfaces like the kettle handle, toilet handle and the phone could not be distinguished. This suggests that the contamination of the door top is unaffected by cleaning behaviours and occurs through deposition of microorganisms over a period of time rather than hand contact. As seen in Fig. 1b the lower touch sites appear have a higher diversity of microorganisms although the significant variability between houses mean that this is not statistically significant.

Two or more sites were positive for *S. aureus* and Gram-negative bacilli in 23% and 63% homes, respectively; these were mostly found on the TV remote and kettle handle which are high hand-touch sites. Gram-negative bacteria included *Pantoea* spp., *Acinetobacter* spp., *Serratia* spp. and a range of pseudomonads. Coliforms such as *Klebsiella pneumoniae* and *Enterobacter cloacae* were recovered from less than 10% of homes. No *Escherichia coli* were isolated. Fungi including Aspergillus spp. and yeasts (mostly Candida spp.) were found on bedroom door top, bedroom windowsill and bedside table, and these sites were also the most heavily contaminated. Logistic regression suggests there is no significant correlation between the presence of fungi and total ACCs (odds ratio − 0.03, 95%CI − 0.06–0.003, p = 0.11). Surprisingly, the sites most likely to yield ‘no growth’ were toilet flush and bathroom door handles. None of the bacterial pathogens identified were multiply resistant to antibiotics.

### Relationships between microorganisms and building and occupant characteristics

**Figure 3:**
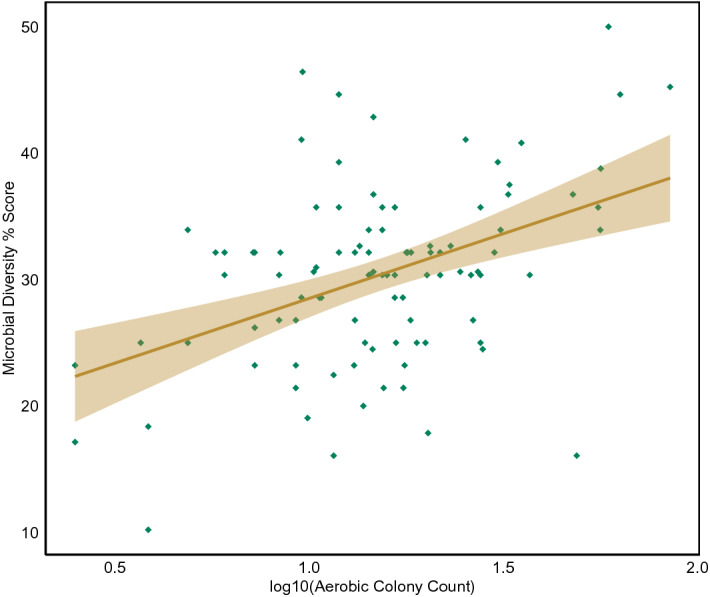
Microbial diversity % score plotted against log_10_ ACC on nutrient agar including a 95% confidence interval.

Regressing microbial diversity with log_10_ACC (Fig. 3) shows a statistically significant positive relationship (F = 22.76, p = 6.415E−06), indicating that surfaces with higher numbers of microorganisms tend to also have a higher number of different species present.

There were no statistically significant relationships between reported window opening frequency (F = 0.13, p = 0.72), trickle vent usage (F = 0.69, p = 0.41), or difference between ventilation type reported (F = 0.947, p = 0.391) and either the total ACC or with ACC at three specific sites in the bedroom: bedside table, window sill and door top. These sites were selected for analysis as they were considered to be the sites which might be most influenced by deposition of microorganisms from the air. Ventilation type was not associated with presence of fungi (p = 0.82). Similarly, there were no statistically significant relationships between pet presence, ventilation type or building age in terms of total ACC or microbial diversity.

**Figure 4:**
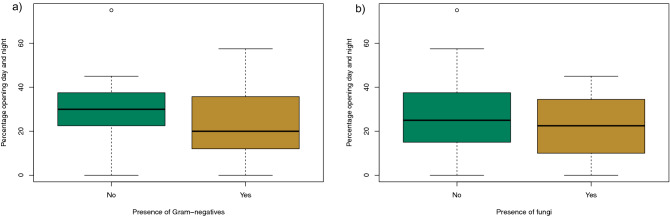
(**a**) Presence of Gram-negatives and (**b**) Fungi vs percentage of window opening day and night combined.

However, there is an association between the frequency of window opening and presence of Gram negative microorganisms (Fig. 4a). Logistic regression was performed on overall percentage of window opening day and night vs whether Gram negatives were reported. The Wald test’s chi-squared value of 18.9 (p = 7.9E−05) suggests that the percentage of window opening is a strongly statistically significant factor in finding Gram negatives. For every unit of opening frequency increase, the chance of finding Gram negatives decreases by 0.97 units (odds ratio 95% confidence interval = 0.94–0.99). Figure 4b shows the same comparison between window opening and the presence of fungi; although there appears to be a qualitative reduction in chance the result is not significant (t = 1.62, p = 0.11).

No significant influence was found of the number of occupants, length of time in the property, age of occupants or pet ownership on either the total ACC or the ACC diversity score. There was a significant difference between ACC in smoker vs non-smoker households (t = 2.468, p = 0.017). Linear regression shows a significant reduction in ACC as the number of smokers increase (F = 4.163, p = 0.044) however, there was no difference in microbial diversity between smoking or and non-smoking households (F = 11.998, p = 0.162). Smokers also had no effect on the presence of fungi (odds ratio 0.77, 95% CI 0.29–1.96, p = 0.55).

**Figure 5:**
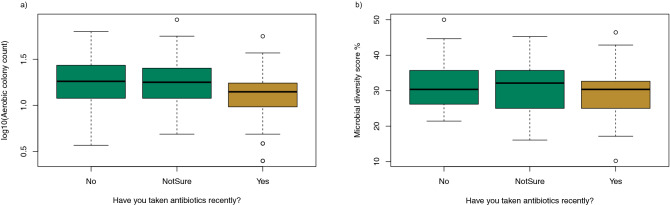
Boxplots of (**a**) microbial diversity and (**b**) log_10_ ACC on nutrient agar in relationship to: “Have you taken antibiotics recently?”.

There was insufficient data to see significant differences in log_10_ACC or ACC diversity scores between categories of usage of antibiotics as described in the survey. However, by grouping responses as Yes or No there is a significant difference in log_10_ACC between groups regarding antibiotic usage (t = 2.51, p = 0.014) as shown in Fig. 5. There was no relationship between antibiotic usage and the presence of fungi (odds ratio 0.99, 95% CI 0.42–2.33, p = 0.55).

**Figure 6:**
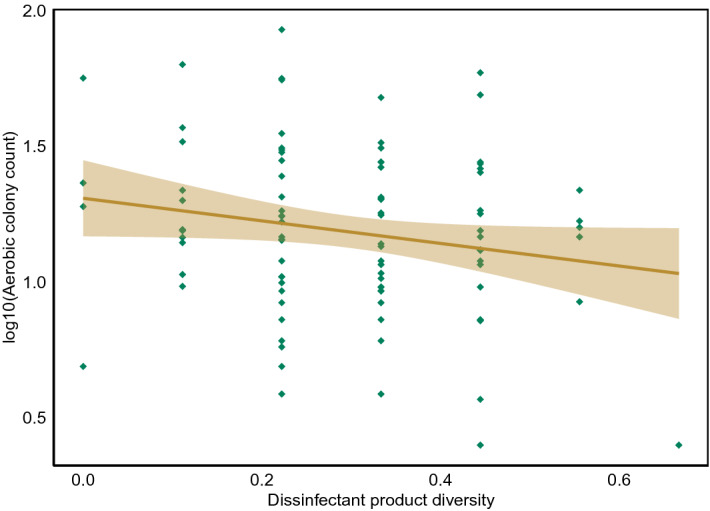
Plot of disinfectant diversity score against log10 ACC including a 95% confidence interval.

Figure 6 shows a linear regression of log_10_ACC compared with disinfectant use mean, which reveals a statistically significant reduction of 0.42 log_10_ACC per 1 unit of disinfectant diversity score increase (F = 3.77, p = 0.05). There is no effect of disinfectant use on microbial diversity score (F = 0.4, p = 0.53) and no statistically significant difference in log_10_ACC between bleach users and non-bleach users (t = 0.07, p = 0.94). There was also no influence of bleach usage on the presence of fungi (odds ratio 1.28, 95% CI 0.52–3.04, p = 0.55).

## Discussion

This study presents a detailed analysis of the relationships between cultivable microorganisms and building and occupant parameters in a sample of 100 homes, predominantly occupied by older adults. It is one of a very small number of studies in a UK context and the first to explicitly look for the presence of pathogens and relate this to the building design and use.

Each of the eight sampled sites revealed its own distinct microbiological character, both in the type and number of cultivable microbes. Human pathogens, particularly *S.aureus*, were more likely to be associated with commonly touched sites such as TV remote, kettle handle and telephone^40^. Whole houses also demonstrated unique microbiological characteristics, with morphologically similar and identifiable microbes observed at multiple sites within the same home^21^. Each home thus displayed its own unique microbiome but with identifiable similarities between other homes according to site.

There was a statistical relationship between homes that opened windows and presence of Gram-negative organisms on sampled sites. This is significant in that it demonstrates a potential effect of window opening on the microbiome and suggests that ventilation design and/or practice may be an important parameter for reducing exposure to specific microorganisms in the home environment. This is likely to be particularly relevant in bedrooms as window opening in these rooms impacts more on long term ventilation rates (i.e. overnight), whereas window opening for thermal comfort will be more short term. It is also possible that bedroom window opening may be an indicator of other health behaviours, for example, occupants who are more health conscious. The presence of both filamentous and yeast-like fungi were not significantly altered by ventilation practices, although the data suggest that fungi were more likely to be found on surfaces if windows are opened infrequently (Fig. 4). Environmental Gram-negative organisms are affected by the use of bleach, so it is also possible that cleaning regimens including the use of disinfectants could confound any effect from ventilation practices.

Despite reports of AMR transmission among households, the study offers some grounds for relief, as there was no evidence for multi-drug resistance among recovered isolates that might have pathogenic potential, i.e. *S. aureus* and human coliforms^39,41^. One important reason for this might be the lack of antibiotic pressures in the home as compared with hospitals. Once this pressure is alleviated by patient discharge, home conditions are unlikely to maintain or drive persistence of MDR organisms unless the patient is immunosuppressed or the organism colonises a major carrier site, e.g. MRSA. None of the latter were found among all recovered *S.aureus*, although it was noted that if one site in the home was positive for *S.aureus*, it was highly likely to find several other sites contaminated, particularly those that are frequently touched. It is possible that eight sites screened per household were insufficient to isolate the full range of viable microorganisms recoverable. Studies similar to this one in the future should include more surface sites in order to gain a more comprehensive view of the range of recoverable organisms on surfaces in peoples’ homes. It should also be possible to cultivate viral organisms as well, which might offer future recommendations for cleaning the home to minimise transmission of colds and flu among inhabitants.

One of the aims of the study was to develop methodologies for assessing the prevalence of pathogens in housing; this has been rarely undertaken, certainly in the UK. There are a range of practical and ethical issues that arise when gathering data in people’s homes and in this case developing a protocol to gather bacteriological samples alongside survey data was an important outcome. The study methodology was able to gather survey data and microbiological samples from the planned number of sites. The original aim had been to target fewer housing development sites with larger numbers of houses. This would have given greater consistency of location and construction systems; however, these types of development were not available within the timescale of the project. Gaining access to homes, in which there is also accurate constructional information, can be difficult. Approaches were made to larger commercial housing providers for older and retired people, but they were not willing to participate in the research. The study was therefore reliant on using housing associations as gatekeepers which narrows the tenure type. The use of more development sites introduced a larger number of variables and controlling these in studies within housing remains a particular challenge. The study developed a sampling strategy to enable the collection of in-situ samples through the use of dipslides on selected sites, using trained personnel in a commercial survey company. This was a cost-effective method and enabled greater number of surveys in shorter periods of time. However, it was important to ensure that clear protocols were in place to facilitate timely transport of samples to the laboratory. This methodology could be applied to larger studies and, given access to facilities for culturing the samples, could be included as a process on other studies that gather data in homes. It could also be undertaken by healthcare professionals in homes, who could also use the building and occupant survey pro-forma for collection of data about the home. We deliberately used microbiology methods analogous to those used in hospital settings to sample microorganisms, as our primary objective was to determine the presence of potential pathogens in a way that is comparable to healthcare studies. Other studies have made use of sequencing techniques^28–33^ which may be a more appropriate methodology where the goal is to characterise the whole of the indoor microbiome. It is recognised that culture-based methods used here have limitations, as they are only able to detect those microorganisms that are both viable and culturable. Sampling efficiency is also a factor that may affect the under reporting of microorganisms, however the dipslides used in this study have been shown to give a comparable recovery to swabbing methods^49^ and contact plates^50^.

The survey data is reliant on reported behaviour. Whilst the results are comparable with other similar studies^9^, it should be noted that some differences were noted between reported and actual behaviour, and actual effects of window opening would be dependent on a range of factors such as door opening and external weather conditions. The study was predominantly carried out during the winter months (Nov-April), however the households may have experienced a range of different weather conditions during this period, which may also influence occupant behaviours such as window opening, use of heating and time spent indoors. It is not possible to evaluate the influence of season on the samples collected, but it is acknowledged that this may influence some microbial species, particularly those associated with environmental sources. Previous microbiome studies have shown relationships between outdoor climate conditions and the species found indoors^29,31^. The sample used in this study was also small, and on relatively new homes, without obvious defects, or problems such as dampness and mould. A further area of research would therefore be on older existing homes, which may have other environmental and bacteriological characteristics, for example, problems of mould growth may lead to increased ill-health and consequent antibiotic use, potentially increasing the antibiotic pressures in the home environment. While we did not find any significant influence of pet ownership it is possible that pets and indeed family demographics such as children and work patterns of adults may affect behaviours in a way that is not captured within the study. Even within the limitations of the study, the research was able to demonstrate an effect of ventilation on the nature and distribution of bacteria within homes. However, other occupancy and behavioural factors, including cleaning habits clearly influence the presence of bacteria in the home, and larger studies are needed to consider how to separate these effects out.

Overall the study was successful in implementing methods and protocols for the collection of survey data and in particular bacteriological samples within homes in a reasonably efficient and cost-effective manner. The key conclusions from the study are:

- The distribution of microorganisms in homes differs between low touch and high touch sites. Low touch locations that are more likely to be contaminated through environmental deposition tend to have higher numbers of microorganisms present and a greater range of different microorganism species.
- Homes tended to show consistent characteristics, with specific microorganisms found at one site likely to be found at several other sites. High use of disinfectants appeared to reduce the diversity of microorganisms found within a home, and both smoking and recent antibiotic use were shown to be correlated to a reduced presence of bacteria.
- Ventilation provision and use has an impact on the presence of Gram-negative bacteria, with increased window opening reducing the likelihood of finding Gram-negative isolates. There is some indication that reduced ventilation also reduced the microbiological diversity, and in the context of a shift to mechanical ventilation, this is of further interest. Greater evaluation of the hygrothermal conditions of homes that contribute to environments that may support pathogens (for example warmer, wetter homes) is also of further interest.
- The data presented here identified a number of microorganisms that could be pathogenic, including *Klebsiella pneumoniae* and *Enterobacter cloacae,* however we found no evidence for home contamination of multidrug resistant pathogens. This may perhaps inform policy about the relative benefits of the home environment in terms of exposure to bacteria, which may be relevant to processes for hospital discharge. However, in this study the homes were selected from a constructional perspective, and recent antibiotic consumption was low. An alternative approach would be to identify households through a clinical route, for example patients being prescribed antibiotics or with chronic health conditions to evaluate conditions in homes where antibiotic use is more prevalent.

## Methods

### Recruitment of homes

**Figure 7:**
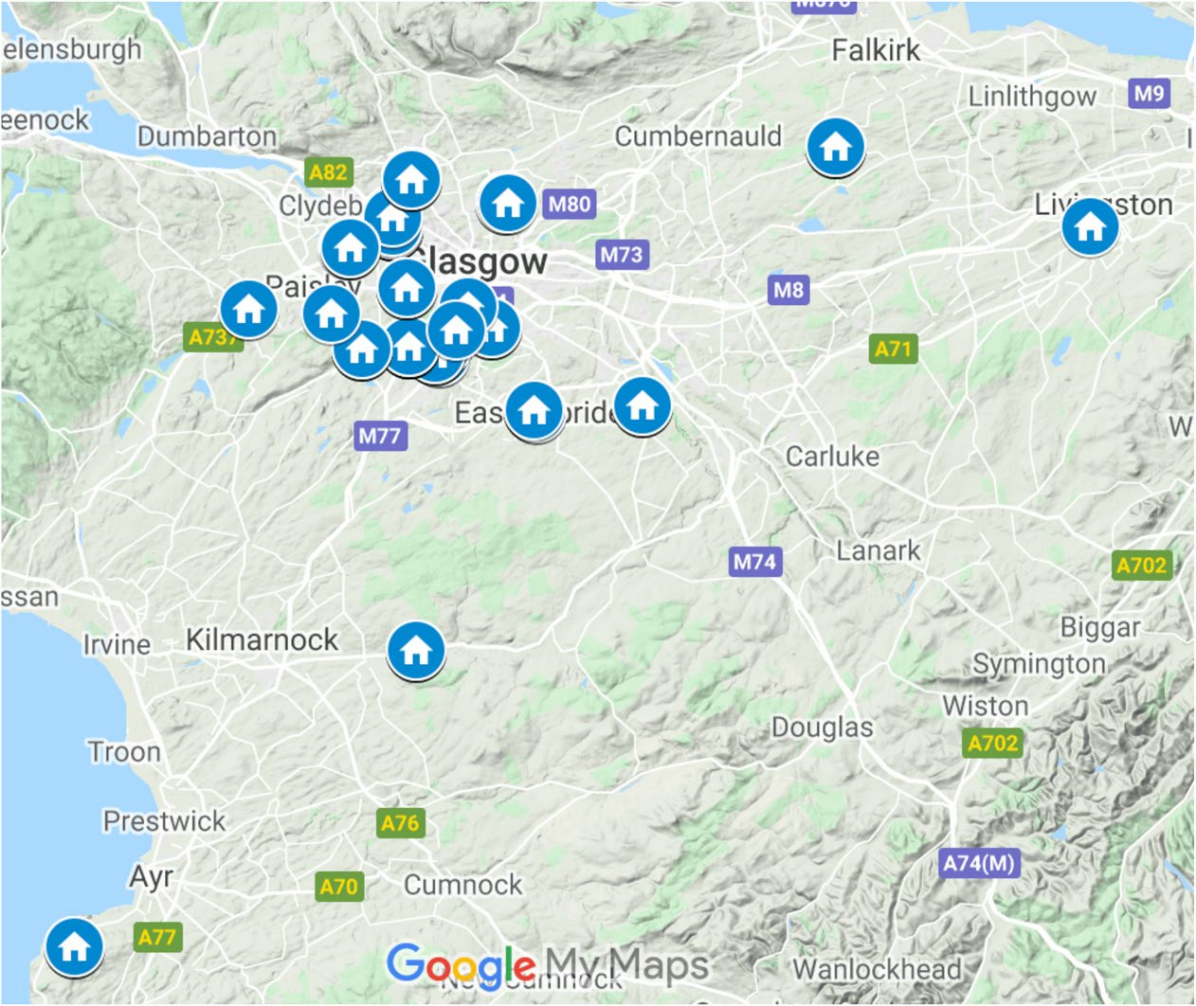
Study dwelling locations (created using Google My Maps: Mapdata©2020).

The study was conducted between November 2017 and April 2018. Households were recruited from seventeen developments across Glasgow, Livingston and Ayr in Scotland UK, covering both urban and rural areas (Fig. 7). Of the 312 households that were initially approached, 109 participated in the occupant survey and 100 agreed to microbial sampling.

The study targeted managed (not sheltered) social housing developments predominately occupied by older people for several reasons. Firstly, older populations are more vulnerable to both environment related health effects and infections and therefore demonstrate increased consumption of antimicrobial agents; consequently, they are also more likely to harbour resistant organisms^42,43^. Secondly, they spend longer periods in the home and so their home environment is important for their health and wellbeing; this also provides more stable conditions for monitoring. Thirdly, the size and nature of this type of accommodation does not vary greatly (similar space standards, occupancy and construction), making comparisons between homes more straightforward.

Several housing associations were approached to identify suitable contemporary housing developments specifically for older people in the Greater Glasgow area. Sites were selected with multiple houses to ease logistical issues of locating and accessing houses and to control for possible confounding variables such as location and weather.

### Occupant survey

The key objective of the survey was to gather a broad range of data across a large number of homes. Letters were distributed by housing associations to tenants of selected developments to provide details of the survey, which was followed up with a house visit from a member of the survey team. In the letter households were advised that in addition to the survey, they would be asked if it is possible to collect environmental samples in their home. Specific details of the microbial sampling (including sampling sites) were not disclosed in advance. The letters also provided tenants with contact details of the survey team, enabling them to opt-out or reschedule ahead of the visit, if required. The door-to-door survey was carried out by a qualified survey company and informed verbal consent was obtained from all participants. An information sheet was provided which included details of the right to withdraw. Experimental protocols were approved by the Glasgow School of Art Research Ethics Committee.

The questionnaire consisted of 55 questions that collected information on household demographics, cleaning and ventilation behaviour, presence of pets, recent hospital exposure, building related factors, general health information and recent antibiotic use. Full details of the survey are provided in Supplementary Information.

The survey data was cross-matched with construction data acquired from the Housing Associations and/or the project architects. This information included dwelling typology and age, orientation, floor area, construction type, energy efficiency measures and ventilation characteristics, including air tightness where this had been measured within a particular group of homes.

### Microbial sampling

At the same time as the household survey, microbial samples were collected in 100/109 homes from eight different surfaces in the home. This was undertaken by the survey team, but training for correct sampling procedures was provided before the study began. Sampling personnel washed hands with soap and water and dried them with a clean disposable towel directly before and after sampling in one home.

A pilot study was undertaken to example the location, nature, replicability and efficacy of sample sites. These needed to be in locations that would be expected to be touched; but also, sites that may be less affected by touch and cleaning that may be more indicative of the overall indoor environment. The sites needed to be consistent across a large number of homes, and also have surfaces onto which a dipslide could be placed. The possibility of collecting dust samples was considered, but this was excluded due the complexity, equipment requirements and additional time required. The sites chosen for screening were: indoor bathroom handle; telephone; kettle handle (kitchen); bedside table; top of bedroom door; TV remote; toilet handle; and bedroom window sill^41^. The site selection deliberately included frequent hand touch sites as well as surfaces such as the bedroom windowsill and top of the bedroom door where microbial contamination would be expected to be related to deposition of microorganisms from the air.

**Figure 8:**
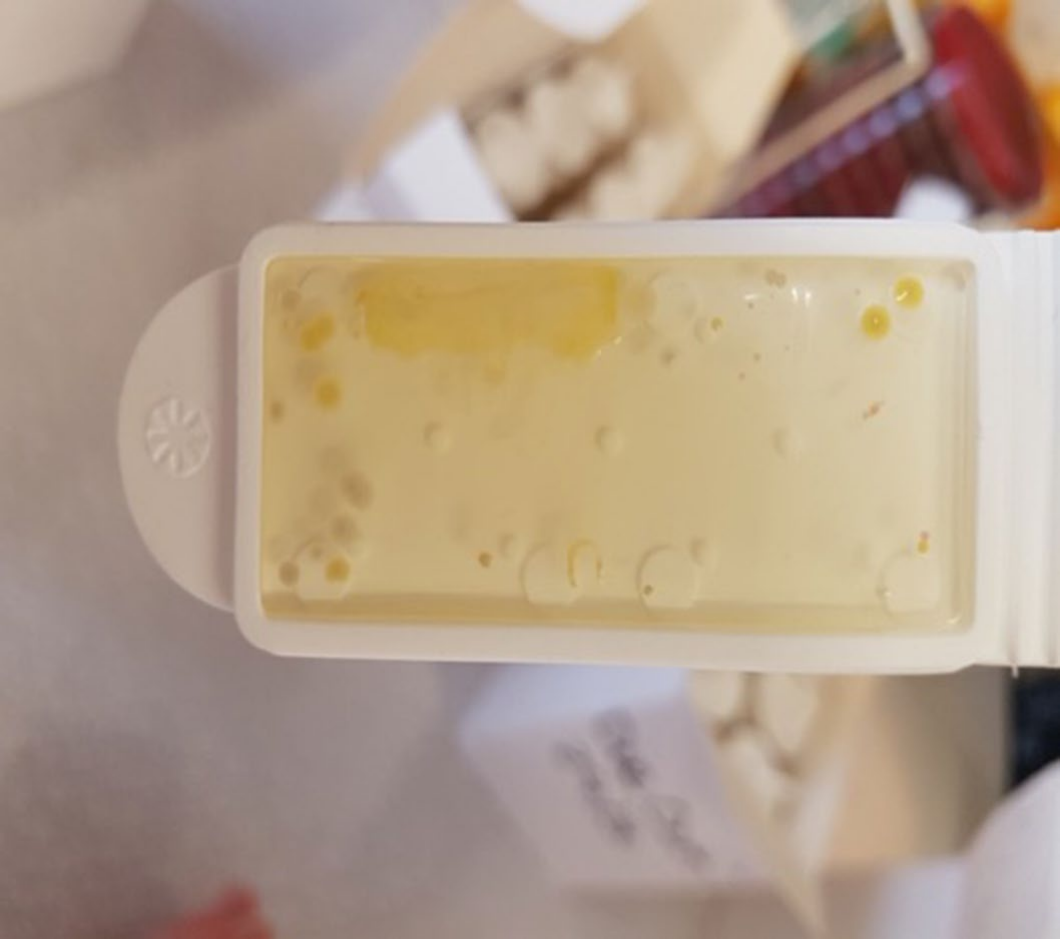
Double-sided dipslide: example from bathroom door handle.

Surfaces were screened using double-sided dipslides coated with nutrient and staphylococcal selective agars (Hygiena Ltd, Watford, UK) to recover total aerobic colony count and an indicator pathogen, Staphylococcus aureus (Fig. 8). These provided quantitative (cfu/cm^2^) and qualitative (MSSA/MRSA) data from hand-touch surfaces^44–47^. S.aureus is the best marker of environmental hygiene in hospitals as well as being the most common cause of bacterial infection worldwide. We also specifically looked for human coliforms from the elemental agar, such as *Escherichia coli* and *Klebsiella pneumoniae*. This was because these organisms have a propensity to be multiply drug resistant in the hospital setting and we wanted to see if any could be recovered from the community. Fungi and yeasts were also readily identified from the elemental agar, but without further identification.

Dipslides were pressed onto chosen sites (if present) for 5–10 s at a pressure of approximately 25 g/cm^2^ without overlap between sampled areas^48^. The slides were replaced in sterile containers and transported to the microbiology laboratory on the day of collection. After loosening caps, the dipslides were incubated for 48 h in air at 35 °C before processing. Sampling was performed in accordance with recognised practices from the Food Standards Agency. Bacteria and fungi were quantified for each site by assessing growth on nutrient agar according to manufacturer’s instructions. Growth on nutrient agar supplied total aerobic colony counts (ACC) per cm^2^ which were classified as follows: no growth (NG) 0 cfu/cm^2^; scanty growth (SG) 2.5 cfu/cm^2^; light growth (LG) 12 cfu/cm^2^; moderate growth (MG) 40 cfu/cm^2^; heavy growth (HG) 100 cfu/cm^2^); and very heavy growth (VGH) 250 cfu/cm^2^. This is comparable to approaches used previously in hospital sampling studies^46–48^. Selective agar highlighted potential coagulase-positive staphylococci, which were sub-cultured onto *Staphylococcus aureus* Identification (SAID) agar (Oxoid Ltd, UK), followed by automated susceptibility testing (VITEK2™) according to routine laboratory protocol. The reader also noted colonial types, morphology and fungi on nutrient agar and performed Gram-stains on a maximum of four cfus per slide, thought to indicate Gram-negative species. Those confirmed as Gram-negative bacilli were screened on UTI selective agar, plated out for purity and identified and characterised by VITEK2, including antimicrobial susceptibility testing.

All methods were carried out in accordance with relevant guidelines and recommendations. The microbial analysis was performed in a CPA accredited clinical laboratory, in accordance with recommendations and standard practices from the Institute of Biomedical Sciences and the Royal College of Pathologists.

### Quantitative analysis

Data from the microbial sampling was combined with occupant survey data for each house. Quantitative analysis of microbial data was carried out using the contamination values indicated above, enabling a mean concentration for ACC across all sites in each house to be calculated. We recorded presence/absence of seven categories of microorganisms identified on the nutrient agar samples: *Staphylococcus* spp.; *Micrococcus* spp.; *Bacillus* spp.; filamentous fungi and yeasts; other Gram positive cocci and rods; Gram negative rods; Gram negative cocci. A diversity measure was calculated to indicate the proportion of these categories present at each site; a diversity of 1 would indicate that all 7 species were present, a diversity of 0 would indicate none.

Household survey data was converted into numerical responses. Questions with a yes–no answer were allocated a value of 1 or 0 respectively, questions with more than one response were given a number for the category. Some additional values were calculated for the analysis based on the survey responses. The questionnaire asked participants how often windows were usually opened in the home during the day and at night, throughout the winter season. Participants responded using a 5-point Likert scale, from ‘Never’ to ‘All the time’ for specific rooms including the kitchen, living room, bedroom(s) and bathroom(s). A total window opening frequency value (%) for winter was calculated by assigning scores to the ordinal data and converting to interval data, using the following weighting: no window/never = 0, monthly = 1, weekly = 2, daily = 3, all the time = 4. A whole house percentage was calculated based on the number of rooms. The value represents the weighted frequency of window opening in the home in winter, during the day and at night. For instance, a value of 0% indicates that windows were reportedly never opened (or no window was present) in all rooms (living room, kitchen, bathroom and bedroom(s)) during the day or night, with 100% indicating all windows were reportedly opened all the time.

The survey asked for information about eight common disinfectant products as well as an additional question that asked about other products used. Almost all of the homes indicated that they had used a disinfectant product or bleach in the last week. To capture the level of disinfectant use, a numerical average was taken of the responses to disinfectant products; a value of 1 indicates the household used 8 different products, a value of 0 indicates none. An additional variable was constructed to indicate whether bleach was used as a yes–no response.

All statistical analysis was carried out using R software (version 4.3). Shapiro–Wilk test was used to examine non-normally distributed microbial counts for ACC on both nutrient agar (p = 2.94E−10) and the selective agar (p = 3.23E−12). Log_10_ transformation returns normally distributed variables (p = 0.44 and p = 0.137 respectively) and hence was used in linear regression analysis. Welch’s t-test was used to compare between means with unequal group variances, and ANOVA enabled assessment of the difference between sample sites. Where appropriate the Kruskal–Wallis test is used normality is not upheld, sample sizes were small. Post-hoc testing using Tukey HSD after ANOVA or Dunn’s test with Holm-Sidak correction after Kruskal–Wallis allowed multiple site comparison where appropriate.

## Supplementary information

Supplementary Information.

Supplementary Information 2.

## Data Availability

Data is available at https://doi.org/10.5518/804.
