# Supplementary material for: Influence of ventilation use and occupant behaviour on surface microorganisms in contemporary social housing
Source: Sci Rep. 2020 Jul 16;10:11841. doi: 10.1038/s41598-020-68809-2 (PMC7366681; doi:10.1038/s41598-020-68809-2)
Supplement: Supplementary file 1 — Supplementary Information. [file 41598_2020_68809_MOESM1_ESM.pdf]

Influence of ventilation use and occupant behaviour on surface microorganisms in contemporary social housing

Sharpe T.[1,] McGill G,[1*] Dancer SJ,[2,3] King M-F,[4] Fletcher L,[4] Noakes CJ.[4]

Affiliations:

[1] Department of Architecture, University of Strathclyde, Scotland, UK

[2] Dept. of Microbiology, Hairmyres Hospital, NHS Lanarkshire, Scotland, UK

[3] School of Applied Sciences, Edinburgh Napier University, Scotland, UK

[4] Water, Public Health and Environmental Engineering Group, School of Civil Engineering, University of Leeds, England, UK

*Correspondence to: grainne.mcgill@strath.ac.uk

Running title: Effect of ventilation on home surface microbes

Keywords: Ventilation; Community; Microbiology; Bacteria; Fungi; Surfaces

Supplementary Information

Table 3: Cultivable Gram-negative bacteria identified from eight sampled sites in Scottish homes.

Most isolates were Pantoea spp. or *Pantoea agglomerans*

Also found:

Acinetobacter baumannii (also lwoffi; radioresistens; ursingii; haemolyticus)

Sphingomonas paucimobilis; Pseudomonas putida (also fluorescens)

Lerclercia adecarboxylata

Paracoccus yeei

Klebsiella pneumoniae

Enterobacter cloacae

Roseomonas gilardii

Aeromonas sobria

Serratia liquefaciens

Brevundimonas spp.

Crononbacter sakazakii

Moraxella spp.
