# Supplementary material for: Influence of ventilation use and occupant behaviour on surface microorganisms in contemporary social housing
Source: Sci Rep. 2020 Jul 16;10:11841. doi: 10.1038/s41598-020-68809-2 (PMC7366681; doi:10.1038/s41598-020-68809-2)
Supplement: Supplementary file 2 — Supplementary Information 2. [file 41598_2020_68809_MOESM2_ESM.pdf]

Influence of ventilation use and occupant behaviour on surface microorganisms in contemporary social housing

[1]Sharpe T. [1]*McGill G, [2,3]Dancer SJ, [4]King M-F, [4]Fletcher L, [4]Noakes CJ.

Affiliations:

[1] Mackintosh School of Architecture, Glasgow School of Art, Scotland, UK

[2] Dept. of Microbiology, Hairmyres Hospital, NHS Lanarkshire, Scotland, UK

[3] School of Applied Sciences, Edinburgh Napier University, Scotland, UK

[4] Water, Public Health and Environmental Engineering Group, School of Civil Engineering, University of Leeds, England, UK

*Correspondence to: grainne.mcgill@strath.ac.uk

Running title: Effect of ventilation on home surface microbes

Keywords: Ventilation; Community; Microbiology; Bacteria; Fungi; Surfaces

Occupancy Information

1. How long have you been living at this property? [*IF DO NOT LIVE HERE – THAND AND CLOSE]

Number of years		Go to Q2

2. How many people live in the house and what are their ages? (*if applicable)

	Description/ name (if given)	Age	
Respondent			Go to Q3
Person 2*			
Person 3*			
Person 4*			
Person 5*			

3. On a typical weekday, how many people are normally in the home: during the day, during the evening and at night?

During the day (8am - 6pm)	Evening (6pm- 12am)	Night (12am- 8am)	
			Go to Q4

4. At the weekend, how many people are normally in the home: during the day, during the evening and at night?

During the day (8am - 6pm)	Evening (6pm- 12am)	Night (12am- 8am)	
			Go to Q5

5. How many bedrooms are there in your home? [WRITE IN NUMBER]

One	1	Go to Q6
Two	2	
Three	3	
Four or more	4	

6. How many people sleep in each of the bedrooms? [WRITE IN NUMBER OF ADULTS AND CHILDREN (UNDER 16). IF NONE WRITE IN '0']

	No. of adults	No. of children	
Bedroom 1 (main)			Go to Q7
Bedroom 2			
Bedroom 3			
Bedroom 4			

7. How many bathrooms are there in the home (including en-suites and wet rooms)?

One	1	Go to Q8
Two	2	
Three or more	3	

8. How many occupants smoke in the home? [ONE ONLY]

One	1	Go to Q9
Two	2	
Three	3	
Four or more	4	
None	5	

9. How often do you dry clothes naturally in the house (for example on radiators or clothes rail) during the winter season? [ONE ONLY]

Every day	1	Go to Q10
Every 2 to 3 days	2	
Once a week	3	
Once a fortnight	4	
Never	5	Go to Q11

10. In which rooms? [ALL THAT APPLY]

Living room	1	Go to Q11
Kitchen	2	
Bathroom(s)	3	
Hallway	4	
Bedroom(s)	5	
Drying cupboard	6	
Other	7	

11. Do you have any house pets? If yes, please provide details.

Yes (please provide details)	Go to Q12
No	Go to Q13

12. If you have a pet, have they ever been prescribed antibiotics?

Yes, in the last month	1	Go to Q13
Yes, in the last 6 months	2	
Yes, in the last year	3	
Yes, more than a year ago	4	
No, never	5	
Can't remember	6	

WINDOW AND DOOR OPENING

13. In winter, how often are the windows usually open in the following rooms in your home during the day? (*if applicable)

	No window	Never	Monthly	Weekly	Daily	All the time	
Kitchen	1	2	3	4	5	6	Go to Q14
Living room	1	2	3	4	5	6	
Main bedroom	1	2	3	4	5	6	
Second bedroom*	1	2	3	4	5	6	
Main bathroom	1	2	3	4	5	6	
Ensuite / wet room*	1	2	3	4	5	6	

14. In winter, how often are the windows usually open in the following rooms in your home during the night? (*if applicable)

	No window	Never	Monthly	Weekly	Daily	All the time	
Kitchen	1	2	3	4	5	6	Go to Q15
Living room	1	2	3	4	5	6	
Main bedroom	1	2	3	4	5	6	
Second bedroom*	1	2	3	4	5	6	
Main bathroom	1	2	3	4	5	6	
Ensuite / wet room*	1	2	3	4	5	6	

15. What are the main reasons for opening windows in your home? [ALL THAT APPLY]

Too warm	1	Go to Q16
To get rid of moisture/ damp	2	
To get rid of smells	3	
To dry clothes	4	
For fresh air / to air the room	5	
It helps me sleep better	6	
For connection to outdoors	7	
Other (please specify)	8	

16. What factors stop you opening the windows in your home? [ALL THAT APPLY]

Don't feel the need to	1	Go to Q17
Pollution	2	
Noise	3	
Security	4	
Heat loss	5	
Insects	6	
Cold draughts	7	
Weather	8	
Can't reach / get to them	9	
Difficult handle/ control	10	
Locked	11	
Other (please specify)	12	

17. Overnight, do you normally keep your bedroom door:

Closed	1	Go to Q18
Open	2	

18. Overnight in your bedroom, do you normally keep curtains/blinds:

Closed	1	Go to Q19
Open	2	

19. During the day in your home, do you normally keep curtains/blinds:

Closed	1	Go to Q20
Open	2	

TRICKLE VENTS

20. [INTERVIEWER – SHOWCARD 01 – TRICKLE VENTS] Are trickle vents installed in your home, and if so, do you know how these are opened / closed?

Yes - Option 1 (underside operated)	1	Go to Q21
Yes – Option 2 (automatic - acoustic/ humidity controlled)	2	
Yes – Option 3 (top operated)	3	
Yes – Option 4 (side operated)	4	
Yes – Other / not sure how they are opened / closed	5	
No trickle vents installed	6	Go to Q24
Not sure if trickle vents installed	7	

21. Do you know if the trickle ventilators are currently opened or closed in the following rooms: (*if applicable)

	No window	No trickle vents present	Opened	Closed	Don't know	
Kitchen	1	2	3	4	5	Go to Q22
Living room	1	2	3	4	5	
Main bedroom	1	2	3	4	5	
Second bedroom*	1	2	3	4	5	
Main bathroom	1	2	3	4	5	
Ensuite / wet room*	1	2	3	4	5	

22. How often do you open or close the trickle vents in your home? [ONE ONLY]

Daily	1	Go to Q24
Weekly	2	
Monthly	3	
Less often	6	
Never	7	Go to Q23

23. Why don't you use the trickle vents? [ALL THAT APPLY]

Didn't know they were there	1	Go to Q24
Don't know how to use them	2	
Can't get to them	3	
Cause draughts	4	
Noise (e.g. blinds rattling or noise from outside)	5	
Worry it will increase heating bills	6	
Don't feel the need to	7	
Other (please specify)	8	

MECHANICAL VENTILATION

24. Is there a mechanical extract fan for ventilation in your bathroom or en-suite?

Yes	1	Go to Q25
No	2	
Not sure	3	

25. Is there a mechanical extract fan for ventilation in your kitchen?

Yes	1	Go to Q26
No	2	
Not sure	3	

***IF NO TO Q24 AND Q25, SKIP TO Q33**

26. If a mechanical ventilation system is installed, does it run continuously?

Yes	1	Go to Q27
No	2	
Not sure	3	

27. Is the mechanical ventilation system(s) currently working / operating?

Yes	1	Go to Q28
No (please explain)	2	
Not sure	3	

28. Have you ever had any problems or concerns relating to your ventilation system:

	Yes	No	
Noise	1	2	IF YES [FOR ANY], Go to Q29<br><br>IF NO, Go to Q30
Cost of running	1	2	
Draughts	1	2	
Performance (stopped working / ineffective)	1	2	
Blocked / dirty	1	2	
Other (please state)	1	2	

29. If yes [FOR ANY OF THE ABOVE], please explain

	Go to Q30

30. Are switches available to boost the ventilation rate in the mechanical ventilation system?

Yes	1	Go to Q31
No	2	Go to Q32
Not sure	3	

31. If switches are available, how often are they used?

A few times a day	1	Go to Q33
Once a day	2	
A few times a week	3	
Once a week	4	
Less than once a week	5	
Never	6	

32. If switches are not available, do you know how the ventilation system is controlled?

Operates automatically when enter the room (PIR/ occupancy sensor)	1	Go to Q33
Operates automatically when humidity/CO_2 levels are high (RH/CO_2 sensor)	2	
Operates automatically when turn on shower/cooker	3	
Other (please state)	4	
Not sure	5	

33. [INTERVIEWER- SHOWCARD 02 – VENTILATION SYSTEMS] Can you identify the type of ventilation system installed in your home?

**Option 1 - continuous mechanical extract ventilation**<br>Air extracted from vents in kitchen and bathroom(s). Fan operates continuously at a low rate.	1	Go to Q34
**Option 2 - passive stack vent**<br>Air extracted from vents in kitchen and bathroom(s) naturally (without a fan / electricity).	2	
**Option 3 - mechanical ventilation with heat recovery**<br>Air extracted from moisture producing rooms & supplied to habitable rooms. System runs continuously and recovers heat from extracted air.	3	
**Option 4 - intermittent extract fans**<br>Air extracted from vents in kitchen and bathroom(s) when needed (does not run continuously).	4	
Other (not listed)	5	
Not sure / No	6	

Indoor environmental quality

34. Have you noticed any mould, mildew or significant condensation on walls or surfaces in the property?

Yes	1	Go to Q35
No	2	Go to Q36

35. If yes, please state where

Living room	1	Go to Q36
Kitchen	2	
Main bedroom	3	
Other bedroom	4	
Bathroom	5	
Other (please state)	6	

36. Do you suspect any humidity/ mould problems inside the floor, walls or ceiling of the home, which are not visible?

Yes	1	Go to Q37
No	2	
Not sure	3	

Perception of the indoor environment

37. Overall, how satisfied are you with the following in your home?

	Very satisfied	Satisfied	Neither/ Nor	Dissatisfied	Very dissatisfied	
Indoor air quality	1	2	3	4	5	Go to Q38
Indoor temperature	1	2	3	4	5	
Natural light levels	1	2	3	4	5	
Noise (from outdoors)	1	2	3	4	5	

Occupant use – cleaning

38. How often are the following activities carried out in the home:

	Never	Less than once a month	Monthly	Weekly	Daily	
Brushing of floors	1	2	3	4	5	Go to Q39
Dusting	1	2	3	4	5	
Vacuuming	1	2	3	4	5	

39. Is your home currently cleaned by any of the following? [ALL THAT APPLY]

Yourself	1	Go to Q40
Family member or friend	2	
Neighbour	3	
Professional cleaning service or cleaner	4	
Management staff	5	
Carer	6	
Other (please specify)	7	

40. In the last year, has the home been cleaned by a professional cleaning service/ cleaner?

Yes	1	Go to Q41
No	2	Go to Q42

41. If yes, please provide details

	Go to Q42

42. Do you use any antibacterial cleaning products or disinfectants in the home?

Flash anti-bacterial wipes	1	Go to Q43
Fairy anti-bacterial washing-up liquid	2	
Dettol antibacterial surface spray	3	
Zoflora dissinfectant	4	
Milton antibacterial surface spray	5	
Cif Disinfectant cleaner	6	
Carex antibacterial hand wash	7	
Tesco anti-bacterial cleaner spray	8	
Other (please state)	9	

43. Please list any other cleaning products that are used routinely in the home (with brand names if possible).

	Go to Q44

44. When was the last time an antibacterial cleaning product / disinfectant was used in the home?

In the last week	1	Go to Q45
In the last month	2	
In the last 6 months	3	
More than 6 months	4	
Never	5	
Can't remember	6	

Surface materials

45. What type of flooring material is there in the following rooms?

	Solid wood	Laminated wood	Tile	Stone	Carpet	Linoleum	PVC floor	Other	
Main bedroom	1	2	3	4	5	6	7	8	Go to Q46
Second bedroom*	1	2	3	4	5	6	7	8	
Living room	1	2	3	4	5	6	7	8	
Kitchen	1	2	3	4	5	6	7	8	
Main bathroom	1	2	3	4	5	6	7	8	
Ensuite/ wet room*	1	2	3	4	5	6	7	8	

46. What type of wall surface is there in the following rooms?

	Paint	Brick	Tile	Stone	Wall-paper	Concrete	Other	
Main bedroom	1	2	3	4	5	6	7	Go to Q47
Second bedroom*	1	2	3	4	5	6	7	
Living room	1	2	3	4	5	6	7	
Kitchen	1	2	3	4	5	6	7	
Main bathroom	1	2	3	4	5	6	7	
Ensuite/ wet room*	1	2	3	4	5	6	7	

General health

47. Do you (or any other occupant) have any of the following health conditions or illnesses? [ALL THAT APPLY]

	Respondent	Person 2	Person 3	Person 4	
Arthritis	1	1	1	1	Go to Q48
Respiratory disease (COPD, asthma or bronchitis; tuberculosis)	2	2	2	2	
Diabetes	3	3	3	3	
Heart disease	4	4	4	4	
Kidney disease	5	5	5	5	
Skin disease	6	6	6	6	
Dementia, Alzheimer's or Parkinsons disease	7	7	7	7	
Mental health problems	8	8	8	8	
Severe stomach, liver or digestive problem	9	9	9	9	
Difficulty seeing	10	10	10	10	
Difficulty hearing	11	11	11	11	
No health conditions	12	12	12	12	
Refused / Don't know	13	13	13	13	
Other health condition (please state)					

48. When was the last time you (or any other occupant) visited a hospital, doctor's surgery or clinical environment?

In the last week	1	Go to Q49
In the last month	2	
In the last 6 months	3	
More than 6 months	4	
Never	5	
Can't remember	6	

49. Please list, if possible, any drugs taken on a regular basis.

	Go to Q50

50. In the past 12 months, have you experienced any of the following symptoms in your home?]

	Yes, often (every week)	Yes, sometimes	No, never	
Dryness of the eyes	1	2	3	Go to Q51
Itchy or watery eyes	1	2	3	
Blocked or stuffy nose	1	2	3	
Runny nose	1	2	3	
Dry throat	1	2	3	
Lethargy and/or tiredness	1	2	3	
Headache	1	2	3	
Dry, itchy or irritated skin	1	2	3	
Other (please state)				

51. Do you believe any of these symptoms to be related to your home environment?

	Go to Q52

Antibiotic use

52. When did you (or any other occupant) last take antibiotics?

In the last month	1	Go to Q53
In the last 6 months	2	
In the last year	3	
More than a year ago	4	
Never	5	Go to Q55
Can't remember	6	

53. Please provide details, if possible, of the antibiotics taken.

	Go to Q54

54. On that occasion, did you complete the full course of antibiotics?

Yes	1	Go to Q55
No	2	

Involvement in monitoring study

55. Would you like to be considered to participate in a detailed monitoring study?

This study will involve physical monitoring of indoor environmental quality and the indoor microbiome during both summer and winter seasons. Monitoring equipment will be left within your home, and collected at the end of the monitoring period. Microbial samples from the air and from surfaces will be collected during setup and collection of monitoring equipment. You will also be asked to take part in an informal interview.

More information on the study is provided in the information sheet. After successful completion of the detailed monitoring study, households involved will receive a shopping voucher worth £50, to thank you for your time. *If yes, collect contact information.

Yes	1
No	2
